# Optimization of bacteriocin production by *Lactobacillus* sp. MSU3IR against shrimp bacterial pathogens

**DOI:** 10.1186/2046-9063-9-12

**Published:** 2013-06-01

**Authors:** Palanisamy Iyapparaj, Thirumalai Maruthiah, Ramasamy Ramasubburayan, Santhiyagu Prakash, Chandrasekaran Kumar, Grasian Immanuel, Arunachalam Palavesam

**Affiliations:** 1 CAS in Marine Biology, Faculty of Marine Sciences, Annamalai University, Parangipettai – 608 502, Tamilnadu, India; 2 Centre for Marine Science and Technology, Manonmaniam Sundaranar University, Rajakkamangalam – 629 502 Kanyakumari District, Tamil Nadu, India; 3 Directorate of Research, SRM University, Kattankulathur – 603 203 Kanchipuram District, Tamilnadu, India; 4 Centre for Ocean Research, Sathiyabama University, Chennai, Tamilnadu, India

**Keywords:** Aquaculture, Bacteriocin, Lactobacillus, Probiotics, Shrimp pathogens

## Abstract

**Background:**

Aquaculture is one amongst the growing and major food producing sectors. Shrimp culture is one of the subsectors of aquaculture that attracts more attention because of the economic interest. However, the shrimp culture systems have been facing severe consequences and economical losses due to disease outbreaks. Risk of disease outbreak can be combated with the application of probiotics. For economically viable production of such probiotic products, the present study provides information on the optimization and partial purification of bacteriocin produced by a goat milk isolate *Lactobacillus* sp. MSU3IR against the shrimp bacterial pathogens.

**Results:**

Bacteriocin production was estimated as a measure of bactericidal activity (arbitrary Unit/ml) over the test strains. The optimum culture conditions and media components for maximum bacteriocin production by *Lactobacillus* sp*.* MSU3IR were: pH: 5.0, temperature: 30°C, carbon source: lactose; nitrogen source: ammonium acetate; NaCl: 3.0% and surfactant: Tween 80. MRS medium was found to extend better bacteriocin production than other tested media. Upon partial purification of bacteriocin, the SDS-PAGE analysis had manifested the presence of two peptide bands with the molecular weight of 39.26 and 6.38 kDa, respectively.

**Conclusion:**

The present results provide baseline trend for the statistical optimization, scale up process and efficient production of bacteriocin by the candidate bacterial strain *Lactobacillus* sp*.* MSU3IR which could be used to replace the usage of conventional chemotherapeutics in shrimp culture systems.

## Background

Aquaculture has become a popular food producing sub sector complement to agriculture. Diseases caused by bacteria and viruses are considered to be an important problem in the intensive rearing of molluscs, finfish, lobster and shrimp [1]. During disease outbreaks, mortality can be as high as 100% [2-5]. Infections caused by pathogenic strains belonging to the species *Aeromonas hydrophila, Vibrio harveyi,V. parahaemolyticus, V.cholerae* and *V. anguillarium* cause traumatic losses in the culture of molluscs, fish and shrimp [2,6-9]. Antibiotics have been widely used to control this problem. In recent years, the therapeutic use of antibiotics against bacterial infection is very much restricted in aquaculture due to its residual effect and development of resistance in bacteria. Hence, the probiotics are extensively used for disease management in aquaculture.

The use of probiotics is prevalent in the aquaculture industry (particularly in shrimp culture) as a means of controlling disease, improving water quality by balancing nutrient (e.g., nitrogen and phosphorus) availability and replacing the use of antibiotics and disinfectants in some cases [10-13]. Probiotics are known to block pathogens by disrupting their virulent gene expression, attachment and cell to cell communication [14]. Probiotic bacteria can also compete with the pathogens for available space and nutrients at host surfaces [15,16]. Many probiotic strains produce antimicrobials, such as lytic enzymes, iron-chelating compounds, antibiotics, hydrogen peroxide, organic acids and bacteriocins [17,18]. Bacteriocins are small peptides that disrupt the integrity of bacterial cell membranes [19,20]. As an alternative tool to control pathogenic bacteria, antimicrobial peptides or bacteriocins are recently being considered. Lactic acid bacteria (LAB) are one of the major resources for bacteriocin biosynthesis.

LAB are comprised of at least ten genera according to taxonomic revisions representing *Aerococcus, Carnobacterium, Enterococcus, Lactobacillus, Lactococcus, Leuconostoc, Pediococcus, Streptococcus, Tetragenococcus* and *Vasococcus*[21]. They are widely used as starter cultures in a variety of food fermentations. It is well known that many lactic acid bacteria show antagonistic activities against other bacteria, including food spoilage organisms and food borne pathogens. There are several different mechanisms responsible for this inhibition. In most cases, the inhibition is caused by the production of organic acid, hydrogen peroxide and bacteriocins [22,23].

Several reports have shown that complex media and well controlled physical factors, such as temperature and pH are required to obtain optimal bacteriocin production [24-27]. Bacteriocin production can be influenced by medium composition and growth phase of microorganism [28]. The production of bacteriocins is usually studied on complex rich media and the most currently evaluated parameters are the concentration of the carbon source, complex nitrogen source and Tween 80 [29,30]. In the light of the above statements, lactic acid bacteria (probionts) and their products (bacteriocins) could be an eco-friendly antimicrobials for substituting the commercial and synthetic antibiotics in aquaculture. However, optimization of culture media for efficient production of bacteriocin that mitigate the growth of shrimp pathogens are under researched. Hence, the present attempt has been undertaken to investigate the influence of various culture conditions and media components on bacteriocin production by *Lactobacillus* sp. MSU3IR.

## Results and discussion

Aquaculture operation alleviates protein shortage and supplies high quality animal to human beings. Also, an environmentally sound and sustainable extensive aquaculture provide employment opportunities and generates income for the people [1]. Shrimp culture scored the major part of the economy across the world. But one of the main obstacles in shrimp culture is disease prevalence. Use of probiotic bacteria to prevent or reduce the risk of diseases is receiving attention as an alternative to antibiotics [11,31,32]. Evaluation of probiotic bacteria capable of producing bacteriocin is becoming an area of rigorous research in several sectors of human nutrition, in animal husbandry and in fish farming [33]. In this context, the present investigation was undertaken to optimize the bacteriocin production by *Lactobacillus* sp. MSU3IR with varying culture conditions and media components for its further application in the field of aquaculture.

### *Lactobacillus* strains

The *Lactobacillus* load of goat milk was ranged from 613.0 ± 6.53 to 96.3 ± 5.73 CFU/ml in 10^-3^ to 10^-5^ dilution respectively [Table 1]. In total, five *Lactobacillus* strains were isolated on MRS agar plates.

**Table 1:** **Enumeration of *Lactobacillus *load (CFU/ml) in Indian goat milk**

**Dilution factor**	**Plate 1**	**Plate 2**	**Plate 3**	**Mean ± SD**
10^-1^	TNTC	TNTC	TNTC	-
10^-2^	TNTC	TNTC	TNTC	-
10^-3^	605	621	613	613.0 + 6.53
10^-4^	311	281	290	294.0 + 12.56
10^-5^	97	103	89	96.3 + 5.73

### Screening of bacteriocin production

The isolated *Lactobacillus* strains were screened for antagonistic activity against indicator strains by the double layer method. Amongst the five strains tested, the candidate bacterium had maximum bioactivity as indicated by the formation of large and clear zone around the colony. With respect to the growth curve, the bacteriocin production by the candidate bacterium was high at the end of stationary phase (48h; data not shown). A similar trend of maximum accumulation of bacteriocin during the stationary phase of growth of *Carnobacterium piscicola* isolated from marine salmonids *Salmo salar* was reported [34,35].

Results indicated that the bioactivity of cell free neutralized supernatant (CFNS) was lost during the treatment with proteinase K, α-chymotrypsin and trypsin whereas catalase had not altered the antagonistic property of CFNS. Thus, it confirmed the presence of bacteriocin in CFNS of candidate bacterium*.* Accordingly, numerous investigators have shown that bacteriocin activity is lost upon treatment with pepsin, trypsin or α - chymo trypsin because of denaturation [22,33,36,37].

**Figure 1:**
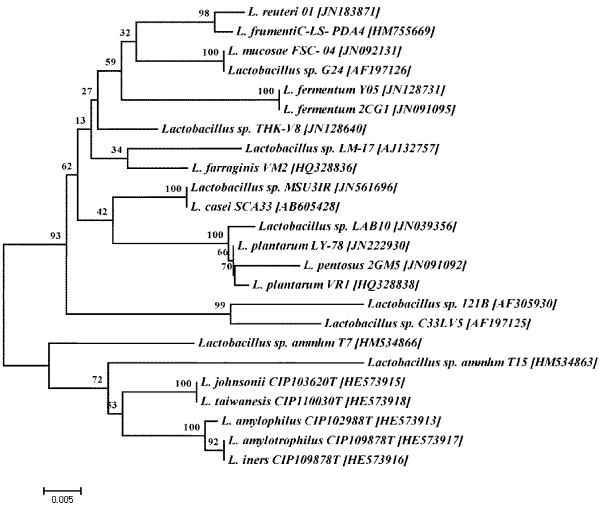
**Phylogenetic tree of the candidate bacterium *Lactobacillus *sp. MSU3IR.**

Furthermore, this potent bacterium was subjected to molecular characterization using 16S rRNA sequencing. The phylogenetic position of candidate bacterium with BLAST analysis inferred 96% similarity to *Lactobacillus* sp. Furthermore, the 16S rRNA sequence of candidate bacterium showed 53.1% GC content with 1371 bp in length and it has been deposited in GenBank [JN561696], NCBI, USA. Phylogenetic analysis revealed that, 16S rRNA sequence of the candidate bacterium has 100% similarity with the existing stain *Lactobacillus casei* AB605428 [Figure 1].

### Optimization of culture condition

Subsequent study was carried out to optimize the bacteriocin production at the end of stationary phase (48h) by *Lactobacillus* sp. MSU3IR and the bacteriocin production was measured in terms of antagonistic activity (AU/ml). The environmental factors, such as pH and temperature has to be optimized for maximum bacteriocin production. Likewise, the bacteriocin production was enhanced by culture conditions optimization in *L. casei*[38] and *Leuconostoc mesenteroides*[39].

**Figure 2:**
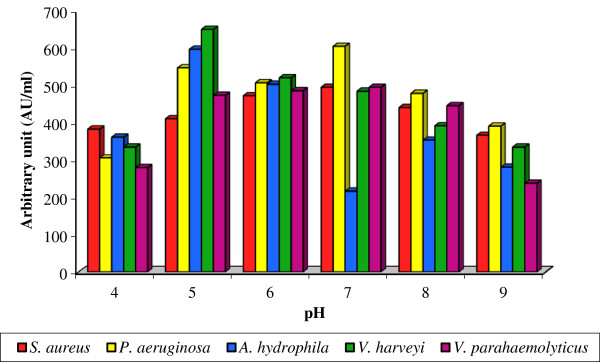
**Effect of various pH on bacteriocin production by *Lactobacillus *sp. MSU3IR.**

Among the tested pH, the maximum bacteriocin production in terms of antagonistic activity was recorded at pH 5.0 and it ranged from 410.4 ± 2.37 to 649.2 ± 5.18 AU/ml. However, further increase in pH found to mitigate the bacteriocin production. The minimum bacteriocin production was recorded at pH 9.0 and it ranged from 238.4 ± 2.34 to 390.4 ± 4.16 AU/ml against the control range of 484.0 ± 3.01 to 604.0 ± 5.63 AU/ml [Figure 2]. Two-way ANOVA revealed that bacteriocin production due to indicator strains is not statistically significant (F: 1.224; P>0.05) whereas it was statistically significant (F: 6.123; P< 0.01) for medium pH. In consonance, the optimum pH for bacteriocin production was usually 5.5 to 6.0 [25,26,40-43]. Comparably the optimum pH for certain bacteriocin production was reported to be less than 5.0 [44-47].

**Figure 3:**
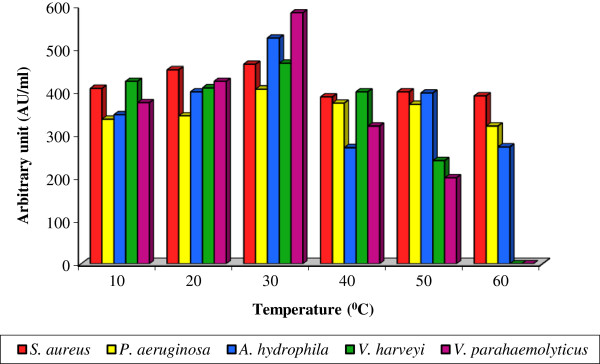
**Effect of various temperature on bacteriocin production by *Lactobacillus *sp. MSU3IR.**

Likewise, the higher bacteriocin production of 464.0 ± 3.13 to 584.0 ± 5.18 AU/ml was recorded at 30°C and further increase in temperature markedly decreased bacteriocin production and the minimum bacteriocin yield was within the range of 272.0 ± 2.29 to 90.4 ± 3.49 AU/ml at 60°C over the control (344.0 ± 2.45 to 473.2 ± 5.47 AU/ml) [Figure 3]. Statistical analysis with two-way ANOVA inferred that the bacteriocin yield was significant due to indicator strains (F: 1.188; P< 0.05) but it was significant (F: 5.479; P< 0.01) for incubation temperature. Similarly, Moonchai et al. [48] also reported that the bacteriocin production by *L. lactis* was optimum at 30°C.

### Optimization of media components

**Figure 4:**
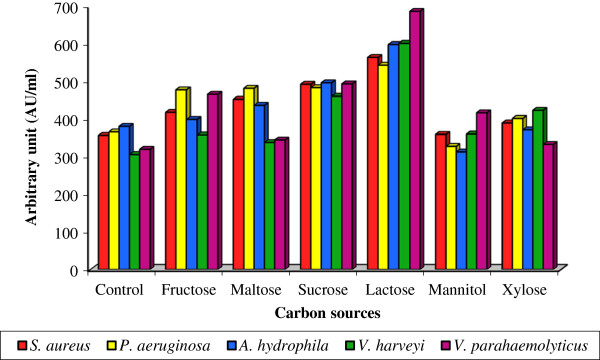
**Effect of various carbon sources on bacteriocin production by *Lactobacillus *sp. MSU3IR.**

Lactose supplementation in culture media favored the maximum bacteriocin yield by *Lactobacillus* sp. MSU3IR in terms of bioactivity ranged from 542.4 ± 3.49 to 685.2 ± 5.90 AU/ml. However, the minimum antagonistic activity (313.2 ± 2.37 to 417.2 ± 3.92 AU/ml) was recorded in mannitol supplied medium over the control value from 306.4 ± 2.65 to 381.2 ± 5.18 AU/ml [Figure 4]. Variation in bacteriocin production due to indicator strains was not statistically significant (F: 0.593; P> 0.05) besides, it was significant (F: 18.504; P< 0.001) for tested carbon sources. In line with our results, Moreno et al. [49] reported maximum bacteriocin yield by *E. faecium* RZS C5 when cultured in MRS supplemented with lactose (5% w/v).

**Figure 5:**
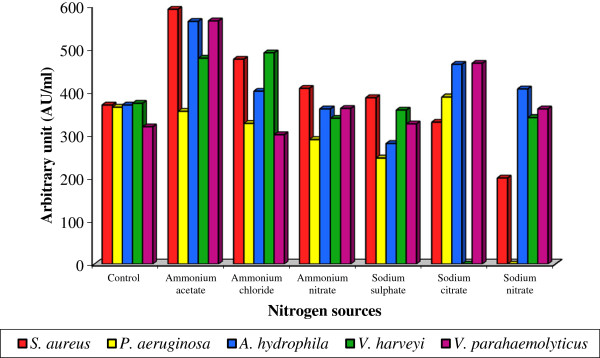
**Effect of various nitrogen sources on bacteriocin production by *Lactobacillus *sp. MSU3IR.**

However the effect of nitrogen source on bacteriocin production by *Lactobacillus* sp. MSU3IR revealed that, ammonium acetate favored the maximum bacteriocin production (354.4 ± 2.65 to 592.0 ± 4.37 AU/ml) and the minimum bacteriocin production was noticed in sodium nitrate (200.0 ± 2.07 to 406.4 ± 3.35 AU/ml) supplied medium over the control (318.4 ± 2.33 to 364.0 ± 3.14 AU/ml) [Figure 5]. Bacteriocin production by the candidate bacterium was statistically significant for indicator strains (F: 1.626; P< 0.05) and nitrogen sources (F: 2.682; P< 0.05). Cell growth and bacteriocin production was shown to be influenced by organic nitrogen source [50]. Accordingly, the present result evidenced that the increment in bacteriocin production was attributed with inorganic nitrogen source.

**Figure 6:**
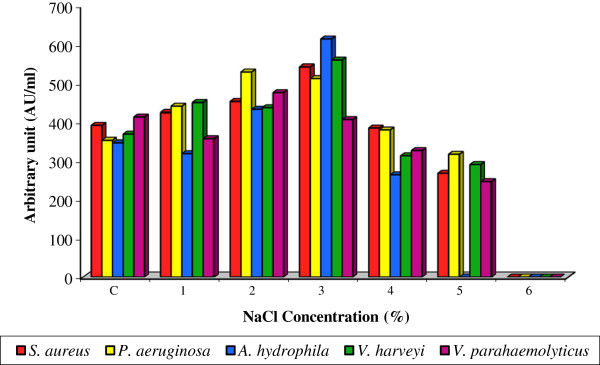
**Effect of various NaCl concentrations on bacteriocin production by *Lactobacillus *sp. MSU3IR.**

The maximum (408.0 ± 3.15 to 614.4 ± 5.00 AU/ml) bacteriocin production by *Lactobacillus* sp. MSU3IR was achieved with 3% NaCl supplementation. However, at 6% NaCl concentration no bioactivity was detected over the control (348.0 ± 2.69 to 414.4 ± 4.25 AU/ml) [Figure 6]. Bacteriocin production due to indicator strains (F: 1.863; P< 0.05) and NaCl concentrations (F: 39.543; P< 0.001) was statistically significant. NaCl could alter the osmolarity of the cell membrane of bacterium which favored the more extrusion of bacteriocin from cell to media. In correlation, Herranz et al. [51] also reported that bacteriocin production by *E. faceium* P13 was high at 3% NaCl and more than 7% of NaCl supplementation reciprocally affected the bacteriocin production.

**Figure 7:**
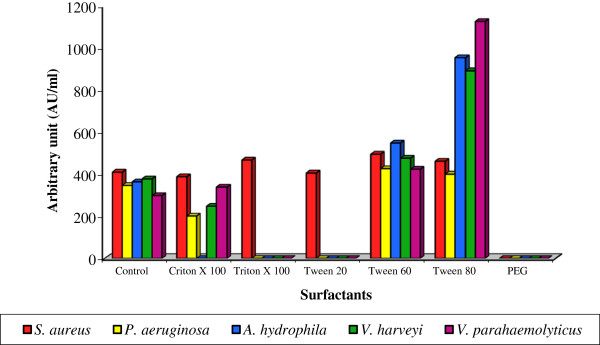
**Effect of various surfactants on bacteriocin production by *Lactobacillus *sp. MSU3IR.**

Bacteriocin production by candidate bacterium emphasized that, the higher bacteriocin yield of 405.2 ± 3.97 to 1126.4 ± 0.63 AU/ml was attained in the medium supplied with Tween 80 compared to other tested surfactants. Incorporation of poly ethylene glycol (PEG) in culture medium was found to terminate the bacteriocin production and expressed no bioactivity than the control (302.4 ± 0.26 to 414.4 ± 4.41 AU/ml) [Figure 7]. Variation in bacteriocin production due to indicator strains (F: 1.043; P<0.05) and surfactants (F: 12.482; P< 0.001) was statistically significant. Similar results were recorded for plantaricin 428 [52], pediocin Actt [53], Lactacin B [54] and Lactocin 705 [38]. Possibly Tween 80 could change the surface tension of the producer cell thereby increasing the rate of bacteriocin release from the cell surface [52].

**Figure 8:**
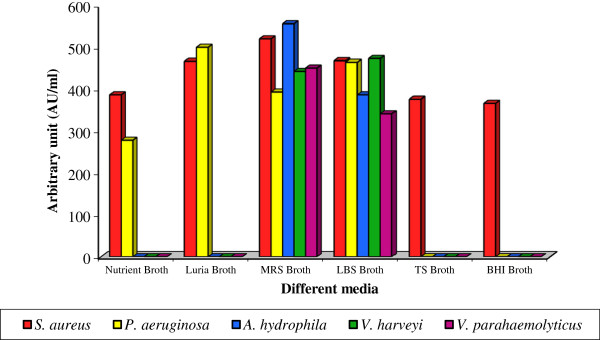
**Effect of various production media on bacteriocin production by *Lactobacillus *sp. MSU3IR.**

To ensure the maximum bacteriocin production, *Lactobacillus* sp. MSU3IR was cultured in various media. The maximum bacteriocin production (393.2 ± 2.61 to 556.0 ± 5.34 AU/ml) was recorded in the control (MRS medium) and followed by *Lactobacillus* selection broth favoured the bacteriocin production (341.2 ± 2.36 to 473.2 ± 3.96 AU/ml). However, the other production media resulted in minimal bacteriocin yield [Figure 8]. Bacteriocin yield due to indicator strains (F: 6.439; P< 0.01) and various media (F: 10.676; P< 0.001) was statistically significant. Earlier reports also evidenced that MRS medium is a better medium for cell growth and bacteriocin production than the other culture media [53,55,56].

### Partial purification of bacteriocin

**Figure 9:**
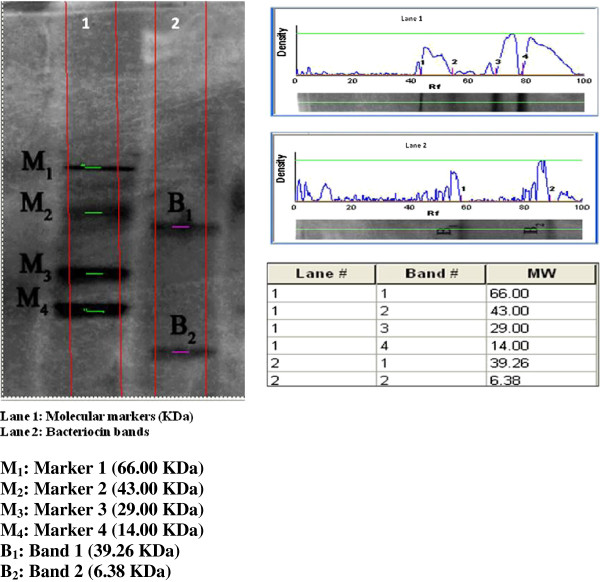
Molecular mass determination of bacteriocin produced by Lactobacillus sp. MSU3IR on comparison with molecular markers in SDS-PAGE.

The baceriocin produced by *Lactobacillus* sp. MSU3IR was partially purified by dialysis and the molecular mass of the bacteriocin was determined by SDS-PAGE analysis. Results inferred that, the bacteriocin preparation contained two distinct bands weighing 39.26 kDa and 6.38 kDa on comparison with the molecular mass of standard markers [Figure 9]. Supportively, the molecular mass of bacteriocin produced by lactic acid bacteria have been reported to fluctuate from 3.40 – 5.60 kDa to 10.00 – 45.00 kDa [57].

## Conclusion

Bacteriocin produced by the candidate bacterium, *Lactobacillus* sp. MSU3IR showed good antagonistic activity against the tested shrimp pathogens. Hence, *ex situ* application of bacteriocin and *Lactobacillus* sp. MSU3IR as probiont in shrimp culture systems are to be studied. The optimization data on bacteriocin production provides basic information for further research on the statistical optimization and industrial scale up process.

## Methods

### *Lactobacillus* strains

For the enumeration and isolation of *Lactobacillus* strains, 1 ml of milk sample was taken and serially diluted (10^-1^ to 10^-5^). From each dilution, 1 ml of sample was taken and pour plated on Man Rogosa Sharpe (MRS) agar plates. After this, the plates were incubated at 37°C for 48 h and the total number of individual viable colonies was counted using a cubic colony counter. Then the morphologically identical colonies were isolated and identified as *Lactobacillus* sp. based on the physical and biochemical characteristics described by Holt et al. [58].

### Shrimp pathogens

Indicator bacterial strains (shrimp pathogens) were collected from the microbial culture collections of Centre for Marine Science and Technology, Manonmaniam Sundaranar University, Kanyakumari District, Tamilnadu, India.

### Screening for bacteriocin production

The isolated *Lactobacillus* strains were screened individually for bacteriocin production by the double layer method as described by Dopazo et al. [59]. For this, the \isolated *Lactobacillus* strains were individually simple streaked on MRS agar plates and incubated at 37°C for 48 h which were overlaid using soft agar (0.8% agar) pre mixed with separate indicator stains. Then the plates were incubated at 37°C for 24 h. The *Lactobacillus* MSU3IR had better antagonistic activity against the indicator strains by forming clear zone of inhibition around it.

The potent strain *Lactobacillus* sp MSU3IR was selected for further study and was found to produce enhanced bacteriocin production in MRS broth at 30°C in 48 h. Hence, the incubation parameters were maintained for analysis to use. The cells were then harvested by centrifugation at 4000 × *g* for 30 min and the culture supernatant was subjected to membrane filtration (0.22 μm). Afterwards, the supernatant was neutralized using 3N NaOH. The resultant cell free neutralized supernatant (CFNS) was treated individually with enzymes, such as proteinase - K, α – chymotrypsin, trypsin and catalase (Sigma, India) at pH 7.0 and temperature 37°C for 2 h in order to check whether the product is bacteriocin. The enzyme activity was terminated by heating the CFNS at 100°C for 10 min and then evaluated for bioactivity [60]. The potent candidate bacterium was characterized using the molecular tool, 16S rRNA sequencing.

### 16S rRNA sequencing

The extraction of genomic DNA of the candidate strain was performed according to the method of Rainey et al. [61]. 16S rRNA gene was amplified using universal primers with the following PCR conditions. The DNA sequence was initially denatured for 5 min at 95°C and annealing of primer to the templates was achieved at 55°C for 30 Sec. Then the samples were maintained at 80°C to allow for hot start conditions and the addition of 5 μl of enzyme solution containing 1 U of *Taq* DNA polymerase in the 1× reaction buffer. PCR was performed with 40 thermal cycles under the standard high-stringency conditions. A 10-min final extension at 72°C was performed at the end of the cycling steps, and then samples were maintained at 4°C. The PCR product was sequenced using the genetic analyzer (Applied bio systems, USA). The comparison of 16S rRNA gene sequence of the candidate strain and the 16S rRNA sequences of other *Lactobacillus* species was done by using national center for biotechnology information – basic local alignment search tool (NCBI-BLAST) database, then the respective gene sequence of the candidate bacterium was deposited in NCBI and the accession number (JN561696) was obtained. The reference gene sequences were retrieved from NCBI GenBank database. All the sequences were aligned using the multiple sequence alignment program CLUSTAL-X 2.0.12 [62]. Phylogenetic tree was constructed using MEGA 4.0 program by following the method of Neighborhood Joining (NJ) described by Saitou and Nei [63].

### Agar well diffusion assay

Bioactivity/production of the bacteriocin by the candidate bacterium was detected using agar well diffusion assay following the method of Tagg and McGiven [64]. In this assay, 25 μl of CFNS was placed on each well of Muller Hinton agar plates which was previously overlaid with approximately 5 ml soft agar (0.8% agar). Soft agar was pre mixed individually with shrimp pathogens that were cultured for 24 h. Then the plates were incubated at 37°C for 24 h and the antagonistic activity in arbitrary unit/ml (AU/ml) was calculated [65] as a measure of bacteriocin production.

$$\mathrm{AU}/\mathrm{ml}=\frac{\mathrm{Diameter}\;\mathrm{of}\;\mathrm{the}\;\mathrm{zone}\;\mathrm{of}\;\mathrm{clearance}\;\left(\mathrm{mm}\right)\times 1000}{\mathrm{Volume}\;\mathrm{taken}\;\mathrm{in}\;\mathrm{the}\;\mathrm{well}\;\left(\mu l\right)}$$

### Composition of production medium

The production medium used in this study is MRS medium and its composition (g/l) is as follows: protease peptone: 10.0, beef extract: 1.0, yeast extract: 5.0, dextrose: 20.0, polysorbate 80: 1.0, ammonium citrate: 20.0, sodium acetate: 5.0, magnesium sulphate: 0.1, manganese sulphate: 0.05 and dipottasium phosphate: 2.0 at a final pH of 6.5 ± 0.20.

### Optimization of culture conditions

The influence of pH on bacteriocin production by the *Lactobacillus* sp. MSU3IR was examined. For this experimental pH, such as 4.0, 5.0, 6.0, 7.0 (control), 8.0 and 9.0 were fixed using 1N NaOH and 1N HCl in the culture medium. Similarly, bacteriocin production with the candidate bacterium was optimized by varying the incubation temperature individually viz., 10, 20, 30 (control), 40, 50 and 60°C. All the flasks were then aseptically inoculated with *Lactobacillus* sp. MSU3IR and kept in an orbital shaker (120 rpm) for 48h. Afterwards, the CFNS was collected from each flask by centrifugation and membrane (0.22μm) filtration. The bacteriocin production in terms of antagonistic activity (AU/ml) was examined against different shrimp pathogens by agar well diffusion assay.

### Optimization of media components

To achieve the maximum bacteriocin production by *Lactobacillus* sp. MSU3IR, the various media components like carbon sources (fructose, maltose, sucrose, lactose, mannitol and xylose individually at 1.0%) and nitrogen sources (ammonium acetate, ammonium chloride, ammonium nitrate, sodium sulphate, sodium citrate and sodium nitrate individually at 1.0%) were substituted in the production medium. Similarly, NaCl at 1.0, 2.0, 3.0, 4.0, 5.0 and 6.0% concentrations and surfactants (Criton X100, Triton X-100, Tween 20, Tween 60, Tween 80 and Poly Ethylene Glycol individually at 1.0%) were supplemented in the production medium. Appropriate control (MRS medium) was also maintained. Then, all the flasks were inoculated aseptically with *Lactobacillus* sp. MSU3IR and kept in an orbital shaker (120 rpm) at 30°C for 48 h. After that the CFNS was collected from each flask and examined for bioactivity to determine the bacteriocin production over shrimp pathogens by agar well diffusion assay.

Likewise, to achieve the maximum bacteriocin production, the *Lactobacillus* sp. MSU3IR was inoculated individually in sterile production medium, such as Nutrient broth, Luria broth, *Lactobacillus* selection broth, Tryptic Soy broth, Brain-Heart infusion broth and MRS broth (control). Then the flasks were incubated at 30°C for 48 h in an orbital shaker (120 rpm). The CFNS was obtained by centrifugation and membrane filtration. Then, the biogenic activity as a measure of bacteriocin production was estimated against indicator strains.

### Partial purification of bacteriocin

Bacteriocin produced by the candidate bacterial strain was purified by the scheme of Bogovic-Matijasic et al. [66]. The candidate bacterium strain was inoculated into the optimized medium and kept under optimum culture conditions. After that the culture was centrifuged at 4000 × g for 30 min at 4°C. Then, the cell free supernatant was precipitated by using 80% ammonium sulphate and settled down by centrifugation at 7000 × *g* for 20 min at 4°C. The pellet containing bacteriocin was suspended in 3 ml of 5 mM sodium phosphate buffer (pH 5.0) and dialyzed against the same buffer for 24 h at 4°C. The retenate was again tested for antagonistic activity against indicator strains to ensure the bioactivity and stored (−20°C) in a sterile container for further analysis. The molecular mass of the dialysed bacteriocin was estimated through SDS-PAGE [67] and gel documentation system (Syngene, UK).

### Statistical analysis

All the experiments were done in six replicates and data obtained in the present study were subjected to statistical analysis, such as two way analysis of variance (ANOVA) using SPSS 16.0 to determine the significant variations between the test groups.

## Abbreviations

LAB: Lactic Acid Bacteria; CFU: Colony-Forming Unit; CFNS: Cell Free Neutralized Supernatant; PEG: Poly Ethylene Glycol; MRS: Man Rogosa Sharpe; NCBI-BLAST: National Center for Biotechnology Information – Basic Local Alignment Search Tool; SDS-PAGE: Sodium Dodecyl Sulfate- Poly Acrylamide Gel Electrophoresis.

## Competing interests

The authors declare that they have no competing interests.

## Authors' contributions

All authors have participated in the research and article preparation. All authors have approved the final article.
